# Vigabatrin Inhibits Seizures and mTOR Pathway Activation in a Mouse Model of Tuberous Sclerosis Complex

**DOI:** 10.1371/journal.pone.0057445

**Published:** 2013-02-20

**Authors:** Bo Zhang, Sharon S. McDaniel, Nicholas R. Rensing, Michael Wong

**Affiliations:** Department of Neurology and the Hope Center for Neurological Disorders, Washington University School of Medicine, St. Louis, Missouri, United States of America; Karolinska Inst, Sweden

## Abstract

Epilepsy is a common neurological disorder and cause of significant morbidity and mortality. Although antiseizure medication is the first-line treatment for epilepsy, currently available medications are ineffective in a significant percentage of patients and have not clearly been demonstrated to have disease-specific effects for epilepsy. While seizures are usually intractable to medication in tuberous sclerosis complex (TSC), a common genetic cause of epilepsy, vigabatrin appears to have unique efficacy for epilepsy in TSC. While vigabatrin increases gamma-aminobutyric acid (GABA) levels, the precise mechanism of action of vigabatrin in TSC is not known. In this study, we investigated the effects of vigabatrin on epilepsy in a knock-out mouse model of TSC and tested the novel hypothesis that vigabatrin inhibits the mammalian target of rapamycin (mTOR) pathway, a key signaling pathway that is dysregulated in TSC. We found that vigabatrin caused a modest increase in brain GABA levels and inhibited seizures in the mouse model of TSC. Furthermore, vigabatrin partially inhibited mTOR pathway activity and glial proliferation in the knock-out mice in vivo, as well as reduced mTOR pathway activation in cultured astrocytes from both knock-out and control mice. This study identifies a potential novel mechanism of action of an antiseizure medication involving the mTOR pathway, which may account for the unique efficacy of this drug for a genetic epilepsy.

## Introduction

Epilepsy is one of the most common neurological disorders and is characterized by recurrent seizures, which may result in significant morbidity and mortality. The first-line treatment for epilepsy is antiseizure medication [1]. While over twenty such medications exist and are effective in many cases, available medications have significant limitations. About one-third of patients with epilepsy are intractable to all medications [1]–[3]. Even when effective, current medications act primarily as symptomatic treatments in suppressing seizures, but do not actually prevent epilepsy [4]. While antiseizure medications target a number of mechanisms of action in the brain, most medications directly inhibit neuronal activity, primarily via modulation of ion channels or neurotransmitter receptors. Although some medications are better for particular types of seizures or epilepsy syndromes, overall all medications are relatively equivalent and non-specific in their efficacy for different types of epilepsy [5]. There are very few, if any, examples of specific targeted therapies for epilepsy with unique effectiveness based on mechanism of action.

Tuberous sclerosis complex (TSC) is one of the most common genetic causes of epilepsy [6], [7]. The seizures in TSC frequently present in childhood, can be of multiple types and are often associated with other neurological problems, such as developmental delay and autism. Infantile spasms, a particularly devastating form of seizures in infants, occur in about one-third of TSC patients. Overall, the majority of patients with TSC and epilepsy have medically-intractable epilepsy [7]. Interestingly, however, seizures in TSC are highly-responsive to the drug, vigabatrin (VGB), with a ∼95% efficacy in stopping infantile spasms in TSC patients [8], [9]. Furthermore, resolution of seizures is often associated with improved developmental progress. Recently it has been proposed that starting VGB at an early age, at or prior to the onset of clinical seizures, may improve the long-term outcome of epilepsy and neurodevelopment in TSC patients [10], [11]. Thus, VGB may represent a rare example of a medication that has specific efficacy for a particular type or cause of epilepsy. VGB is known to have antiseizure effects by elevating brain gamma-aminobutyric acid (GABA) levels via inhibition of its breakdown by GABA transaminase [12]–[14]. However, since VGB and other GABA-modulating drugs are not as effective in other types of epilepsy, whether this or some other mechanism accounts for VGB's unique effectiveness for seizures in TSC is poorly understood.

In addition to epilepsy, developmental delay, and autism, TSC is characterized by the tendency to form tumors in the brain and other organs [15]. Recently, significant advances in understanding the genetics and molecular pathophysiology of TSC have been made, which largely explain the mechanistic basis of tumorigenesis in this disease. Two genes, *TSC1* and *TSC2*, cause TSC and encode the proteins, hamartin and tuberin, respectively, which bind together to form a functional complex that inhibits the mammalian target of rapamycin (mTOR) pathway. As the mTOR pathway is normally involved in stimulation of cell growth and proliferation, mutation of one of the TSC genes leads to disinhibition of the mTOR pathway, which promotes excessive cell growth and tumor formation in TSC. The mTOR pathway, particularly mTOR complex 1 (mTORC1), is involved in a variety of other functions and has also been implicated in epileptogenesis in TSC [16], [17]. mTORC1 inhibitors are proven therapies for tumor growth in TSC [18] and are being investigated as treatments for epilepsy and other neurological complications of TSC. Our novel hypothesis is that vigabatrin has its unique efficacy for seizures in TSC by modulating the mTOR pathway. In this study, we examined the effect of VGB on epilepsy in a mouse model of TSC, including possible interactions with the mTOR pathway.

## Materials and Methods

### Ethics Statement

Care and use of animals were conducted according to an animal protocol approved by the Washington University Animal Studies Committee (IACUC #A-3381-01, Approval #2010-0235). All efforts were made to minimize animal discomfort and reduce the number of animals used.

### Animals and drug treatment

*Tsc1* ^flox/flox^-GFAP-Cre knock-out (*Tsc1* ^GFAP^CKO) mice with conditional inactivation of the *Tsc1* gene predominantly in glia were generated as described previously [19]. *Tsc1* ^flox/+^-GFAP-Cre and *Tsc1* ^flox/flox^ littermates have previously been found to have no abnormal phenotype and were used as control animals in these experiments.

Three-week-old *Tsc1* ^GFAP^CKO mice were treated with vehicle (saline) or VGB at different doses (50, 100, 200 mg/kg/day, i.p) for one week for Western blot and GABA concentrations, for four weeks (200 mg/kg/day) for histology and immunohistochemistry analysis, and for up to 10 weeks (200 mg/kg/day) for video-EEG monitoring. Three weeks of age is just prior to the previously-documented onset of seizures and pathological abnormalities in *Tsc1* ^GFAP^CKO mice [19]–[21]; thus this protocol has the potential to prevent the onset of neurological abnormalities in these mice, as previously demonstrated for rapamycin [21]. The dosing for the histology and video-EEG experiments were selected to maximize the chance of detecting an effect based on the western blot experiments, as well as on clinically-relevant dosing. Vehicle-treated non-KO littermates served as additional controls. Other vehicle or VGB-treated *Tsc1* ^GFAP1^CKO mice and control mice were monitored for body and brain weight measurements (for up to 4 weeks) or for survival analysis.

### Video-electroencephalography monitoring

Vehicle- and VGB-treated *Tsc1* ^GFAP^CKO mice underwent weekly video-EEG monitoring starting at 3 weeks of age, using established methods for implanting epidural electrodes and performing continuous video-EEG recordings, as described previously [20], [21]. Briefly, mice were anesthetized with isoflurane and placed in a stereotaxic frame. Epidural screw electrodes were surgically implanted and secured using dental cement for long term EEG recordings. Four electrodes were placed on the skull: one right and one left central electrodes (1 mm lateral to midline, 2 mm posterior to bregma), one frontal electrode (0.5 mm anterior and 0.5 mm to the right or left of bregma) and one occipital electrode (0.5 mm posterior and 0.5 mm to the right or left lambda). The typical recording montage involved two EEG channels with the right and left central “active” electrodes being compared to either the frontal or occipital “reference” electrode. Video and EEG data were acquired simultaneously with an XLTEK video-EEG system. Forty-eight-hour epochs of continuous video-EEG data were obtained once a week from each mouse, for the life of the animal, and were analyzed for seizures. Electrographic seizures were identified by their characteristic pattern of discrete periods of rhythmic spike discharges that evolved in frequency and amplitude lasting at least 10 seconds, typically ending with repetitive burst discharges and voltage suppression. On video analysis, the behavioral correlate to these seizures typically involve head bobbing, rearing with forelimb clonus, and occasional generalized convulsive activity. Seizure frequency (number of seizures per 48-hour period, based on analysis of the entire EEG record) was calculated from each 48-hour epoch.

### Western blotting

After one week of VGB or vehicle treatment, western blot analysis was used to measure the ratio of phospho-S6 (P-S6, Ser240/244) and total S6 in the neocortex and hippocampus of *Tsc1* ^GFAP^CKO mice, as an assessment of mTOR pathway activity. Western blotting was performed using standard methods as described previously [21]. Briefly, neocortex and hippocampus were dissected and homogenized separately. Equal amounts of total protein extract were separated by gel electrophoresis and transferred to nitrocellulose membranes. After incubating with primary antibodies to P-S6 (Ser240/244), S6, or beta-actin (1∶1,000, Cell Signaling Technology, Beverly, MA), the membranes were reacted with a peroxidase-conjugated secondary antibody (Cell Signaling Technology). Signals were detected by enzyme chemiluminescence (Pierce, Rockford, IL) and quantitatively analyzed with ImageJ software (NIH, Bethesda, MD).

### Measurement of brain GABA concentration in *Tsc1* ^GFAP^CKO mice

After one week of VGB or vehicle treatment, brain GABA concentrations were measured using a mouse gamma-aminobutyric acid (GABA) ELISA kit (Novatein Biosciences, Cambridge MA; sensitivity 2.5–80 ng/ml) according to the manufacturer's instructions. One week of VGB treatment was used before assaying GABA levels, which should start to increase within one day and reach a steady state after a few days of VGB treatment based on previous studies (14). Briefly, *Tsc1* ^GFAP^CKO mice and non-KO littermates were treated with VGB (200 mg/kg/day) or vehicle (saline) for one week. Neocortex and hippocampus were dissected and homogenized separately and a centrifuged supernatant of each sample was used to measure the GABA level by using the GABA ELISA kit. The protein concentration of each supernatant was determined using a BCA protein assay (Thermoscientific, Rockford, IL).

### Histology/Immunohistochemistry

After four weeks of VGB or vehicle treatment, histological analysis was performed to assess glial proliferation and neuronal organization by standard methods, as previously described [21]. Four weeks of VGB treatment was used based on the expected time course to detect differences in these histological assays from previous studies [19], [21]. In brief, brains were perfusion-fixed with 4% paraformaldehyde and cut into 50 µm sections with a vibratome. Some sections were stained with 0.5% cresyl violet. Other sections were labeled with GFAP antibody (anti–rabbit; 1∶500; Sigma) and then rhodamine-conjugated anti–rabbit IgG (1∶500; Sigma, St. Louis, MO). Images were acquired with a Nanozoomer HT system (Hamamatsu, Bridgewater, NJ). In images from coronal sections at approximately 2 mm posterior to bregma and approximately 1 mm from midline, regions of interest were marked in neocortex by a 200 µm-wide box spanning from the neocortical surface to the bottom of layer VI and in hippocampus by a 200×200 µm^2^ box within the striatum radiatum of CA1 and dentate gyrus. GFAP-immunoreactive cells were quantified in the regions of interest from two sections per mouse from a total of four to five mice per group.

### Astrocyte culture and mTOR activity in vitro

Astrocytes were obtained from mixed cell cultures of the forebrains as described previously [22]. The method was slightly modified to remove microglial cells completely. Briefly, the forebrains of newborn *Tsc1* ^GFAP1^CKO mice and non-KO littermates were dissected and the dissociated brain cells were seeded in a poly-D-lysine-coated 75 cm^2^-culture flask (Becton Dickinson Labware, Franklin Lakes, NJ). Cells were cultured for 8–10 days until being confluent, then they were vigorously hand-shaken for 0.5–1 min to remove microglial cells and O-2A lineage cells that were present on the astrocyte monolayer, followed by medium exchange and incubation overnight in a CO_2_ incubator. The purification procedure was repeated three times during the subsequent 3 days. Finally, the flask was washed with DMEM several times and the medium were changed to Neurobasal medium for 6 hours before treatment.

VGB at a concentration of 0.06, 0.3 or 0.6 mM or phenobarbital at 0.01, 0.1, and 1 mM was added to the medium and incubated for 16 hours. VGB dosing was based on previous in vitro physiology studies on inhibiting GABA transaminase [23], [24] and phenobarbital dosing was based on potentiation of GABA inhibition in neurons and related pharmacological effects on astrocyte cultures [25]–[27]. Samples were collected after trypsinization with 0.25% trypsin-EDTA (Invitrogen, Grand Island, NY), and then western blotting analysis was done to measure the ratio of phospho-S6 (P-S6) and total S6 as described above.

### Statistics

All statistical analysis was performed using GraphPad Prism (GraphPad Software). Quantitative differences between groups were analyzed by one-way ANOVA with Tukey's multiple comparisons post hoc tests when comparing one factor over more than two groups or by repeated measures two-way ANOVA when comparing multiple treatment variables (e.g. effect of treatment and genotype). Comparable non-parametric tests were used when data did not fit a normal distribution. Chi-Square test was used for survival analysis. Quantitative data are expressed as mean ± SEM. Statistical significance was defined as p<0.05.

## Results

### VGB treatment decreased the development of seizures and modestly improved survival in *Tsc1* ^GFAP^CKO mice

**Figure 1:**
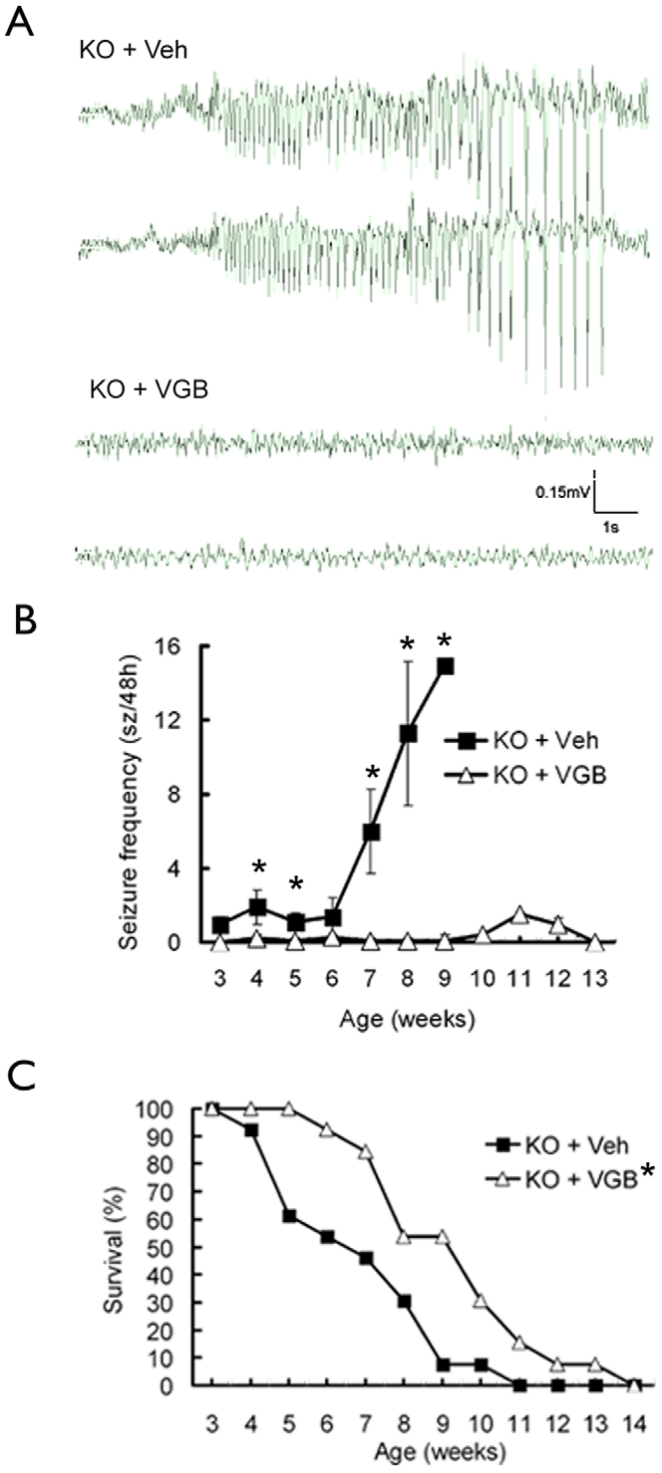
VGB treatment inhibited seizures and moderately improved survival in *Tsc1* ^GFAP^CKO mice. (A) Representative EEG recordings of *Tsc1* ^GFAP^CKO mice treated with vehicle or vigabatrin. (B) Seizures started to develop in vehicle-treated *Tsc1* ^GFAP^CKO mice (Fig. 1A, KO + Veh) around 3 weeks and became progressively more frequent with age. VGB treatment (KO + VGB) almost completely suppressed the development of seizures in *Tsc1* ^GFAP^CKO mice (*p<0.05 by one-way ANOVA, n = 13 mice/group). (C) Survival analysis showed that vehicle-treated *Tsc1* ^GFAP^CKO mice die prematurely with 50% mortality around 7 weeks of age and 100% mortality by 11 weeks. VGB treatment modestly improved the survival of *Tsc1* ^GFAP^CKO mice compared to the vehicle treated *Tsc1* ^GFAP^CKO mice (*p<0.05 by Chi-Square test, comparing the two groups, n = 13 mice/group), but all VGB-treated mice still died by age of 14 weeks. KO = *Tsc1* ^GFAP^CKO mice, Veh  =  vehicle, VGB  =  vigabatrin.

*Tsc1* ^GFAP^CKO mice develop a progressive epilepsy starting around 4 weeks of life and then die prematurely by 3 months of age [19]–[21]. Consistent with previous studies, video-EEG showed that vehicle-treated *Tsc1* ^GFAP^CKO mice developed seizures at around 3–5 weeks of life (1.0±0.4 seizures/48 hours), which became progressively more frequent with age (11.3±3.9 seizures/48 hours at 8 weeks). In contrast, VGB treatment (200 mg/kg/day) almost completely suppressed seizures in *Tsc1* ^GFAP^CKO mice, as monitored by video-EEG between 3 and 13 weeks of age (0.0±0.0 and 0.1±0.1 seizure/48 hours at 3 and 8 weeks of age, respectively; Fig 1A,B). Eleven out of thirteen mice were found to have seizures in vehicle- treated group while only seven out of thirteen VGB-treated *Tsc1* ^GFAP^CKO mice had seizures. Survival analysis confirmed previous studies demonstrating that vehicle-treated *Tsc1* ^GFAP^CKO mice die prematurely, with 50% mortality around 7 weeks of age and 100% mortality by 11 weeks. VGB treatment caused a modest, but significant, increase in survival of *Tsc1* ^GFAP^CKO mice compared to vehicle-treated *Tsc1* ^GFAP^CKO mice (Fig 1C). However, all VGB-treated mice still died by 14 weeks of age.

### VGB treatment did not affect the brain and body weight of *Tsc1* ^GFAP^CKO mice

Consistent with previous studies [19], [21], vehicle-treated *Tsc1* ^GFAP^CKO mice developed dramatic, diffuse megalencephaly (brain weight = 501±9 mg at 7 weeks of age) compared with non-KO control mice (416±3 mg; p<0.05). VGB treatment at dose of 200 mg/kg/day for four weeks did not prevent the megalencephaly in *Tsc1* ^GFAP^CKO mice (489±4; p<0.05 compared with non-KO control mice). VGB treatment also had no significant effect on body weight of *Tsc1* ^GFAP^CKO mice. At 7 weeks of age, there was a trend towards a decrease in body weight in vehicle-treated *Tsc1* ^GFAP^CKO mice (16.2±0.7 g) compared with non-KO control mice (19.1±0.8 g) and an increase with VGB treatment in *Tsc1* ^GFAP^CKO mice (18.5±1.20 g), but these were not statistically significant. Furthermore, there was no obvious effect of VGB on behavior or activity.

### VGB treatment increased brain GABA concentration in *Tsc1* ^GFAP^CKO and control mice

**Figure 2:**
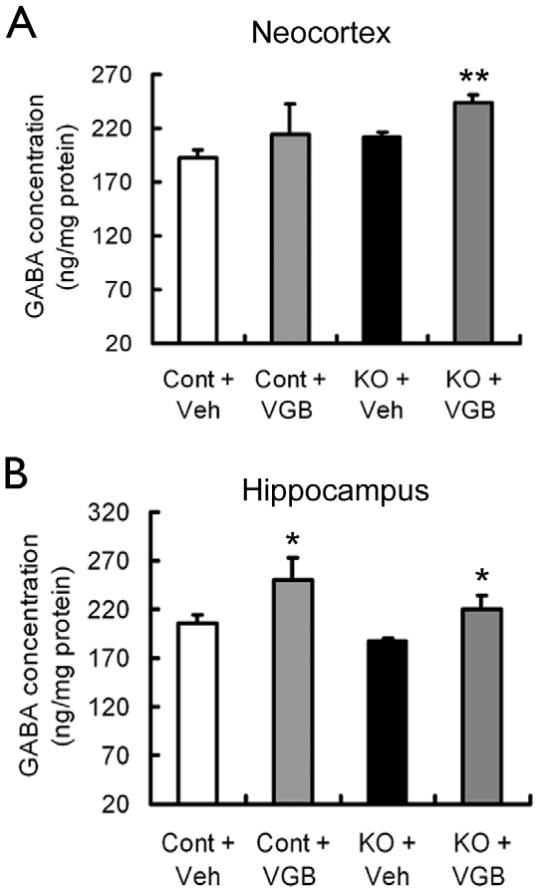
VGB treatment increased brain GABA concentrations in *Tsc1* ^GFAP^CKO and control mice. GABA levels were measured using a commercial mouse GABA ELISA kit. VGB treatment for one week increased the GABA levels in both neocortex (A) and hippocampus (B) of *Tsc1* ^GFAP^CKO mice (KO+VGB), compared with the vehicle-treated *Tsc1* ^GFAP^CKO group (KO+Veh). Similar effects of VGB were observed in control mice in hippocampus only (Cont+VGB versus Cont+Veh). Data were derived from three separate experiments. *p<0.05, **p<0.01 versus vehicle-treated mice by two-way ANOVA (n = 6–7 mice/group). Cont  =  control mice, KO  =  *Tsc1* ^GFAP^CKO mice, Veh  =  vehicle, VGB  =  vigabatrin.

Brain gamma-aminobutyric acid (GABA) concentrations in neocortex and hippocampus were measured using a mouse GABA ELISA kit. GABA levels in neocortex (Fig. 2A) and hippocampus (Fig. 2B) were similar in vehicle-treated control and *Tsc1* ^GFAP^CKO mice. VGB treatment for one week increased GABA levels in neocortex and hippocampus of *Tsc1* ^GFAP^CKO mice and hippocampus of control mice.

### VGB treatment decreased the number of GFAP positive cells in hippocampus of *Tsc1* ^GFAP^CKO mice

**Figure 3:**
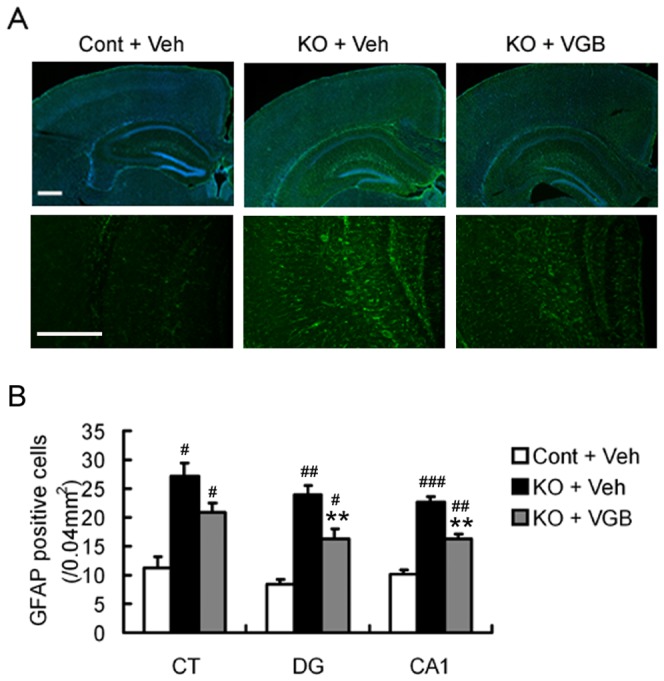
VGB treatment decreased the number of GFAP positive cells in hippocampus of *Tsc1* ^GFAP^CKO mice. (A) Vehicle-treated *Tsc1* ^GFAP^CKO mice (KO + Veh) displayed a diffuse increase in GFAP-positive cells in neocortex and hippocampus compared with the vehicle-treated control mice (Cont + Veh). VGB treatment partially prevented this increase in GFAP-positive cells in *Tsc1* ^GFAP^CKO mice (KO + VGB). (B) Quantitative analysis demonstrated a 2–2.5 fold increase in GFAP-positive cell in vehicle-treated *Tsc1* ^GFAP^CKO group (KO + Veh) compared with vehicle-treated control group (Cont + Veh) in neocortex (CT), dentate gyrus (DG) and CA1 of hippocampus. #p<0.05, ## p<0.01, ### p<0.001 versus vehicle-treated control mice by two-way ANOVA (n = 4–5 mice/group). VGB treatment decreased GFAP-positive cells in *Tsc1* ^GFAP^CKO mice (KO+VGB). **p<0.01 versus vehicle-treated *Tsc1* ^GFAP^CKO mice by two-way ANOVA (n = 4–5 mice/group). Scale bars = 500 µm. Cont  =  control mice, KO  =  *Tsc1* ^GFAP^CKO mice, Veh  =  vehicle, VGB  =  vigabatrin, CT  =  neocortex, DG  =  dentate gyrus, CA1  =  CA1 pyramidal cell layer of hippocampus.

*Tsc1* ^GFAP^CKO mice exhibit a progressive, diffuse increase in GFAP-positive astrocytes related to excessive glial proliferation [19], [21]. Consistent with previous studies, vehicle-treated *Tsc1* ^GFAP^CKO mice showed a large increase in GFAP-immunoreactive cells compared with non-KO control mice (Fig. 3A, B). VGB treatment caused a significant decrease in the number of GFAP positive cells in hippocampus of *Tsc1* ^GFAP^CKO mice.

### VGB treatment did not prevent neuronal disorganization in *Tsc1* ^GFAP^CKO mice

**Figure 4:**
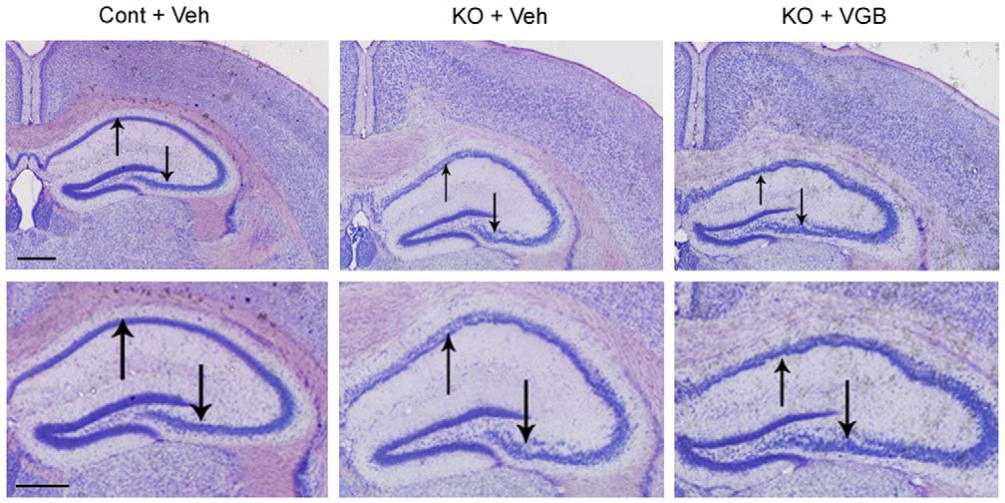
VGB treatment did not prevent neuronal disorganization in *Tsc1* ^GFAP^CKO mice. The left panels show Cresyl violet staining of a brain section at low (upper) and high (lower) magnification of vehicle-treated control mice (Cont + Veh). Vehicle-treated *Tsc1* ^GFAP^CKO group (KO + Veh) exhibited widely dispersed pyramidal cell layers (arrows in the middle panels) in all regions of hippocampus (CA1–CA4) compared with control mice. As shown in the right panels, VGB treated *Tsc1* ^GFAP^CKO mice (KO + VGB) had a similar pattern as vehicle-treated *Tsc1* ^GFAP^CKO group, with no apparent effect on this neuronal disorganization (arrows in right panel). Scale bar = 500 µm. Cont  =  control mice, KO  =  *Tsc1* ^GFAP^CKO mice, Veh  =  vehicle, VGB  =  vigabatrin.

*Tsc1* ^GFAP^CKO mice exhibit disorganization and dispersion of the pyramidal cell layer of hippocampus [19], [21]. Consistent with previous studies, Cresyl violet staining demonstrated that vehicle-treated *Tsc1* ^GFAP^CKO mice had widely dispersed pyramidal cell layers (Fig. 4, arrows in the middle panels) in all regions of hippocampus (CA1–CA4) compared with control mice. VGB treatment had no apparent effect on this neuronal disorganization in *Tsc1* ^GFAP^CKO mice (Fig. 4, arrows in right panels).

### VGB treatment decreased activation of the mTOR pathway *in vivo*

**Figure 5:**
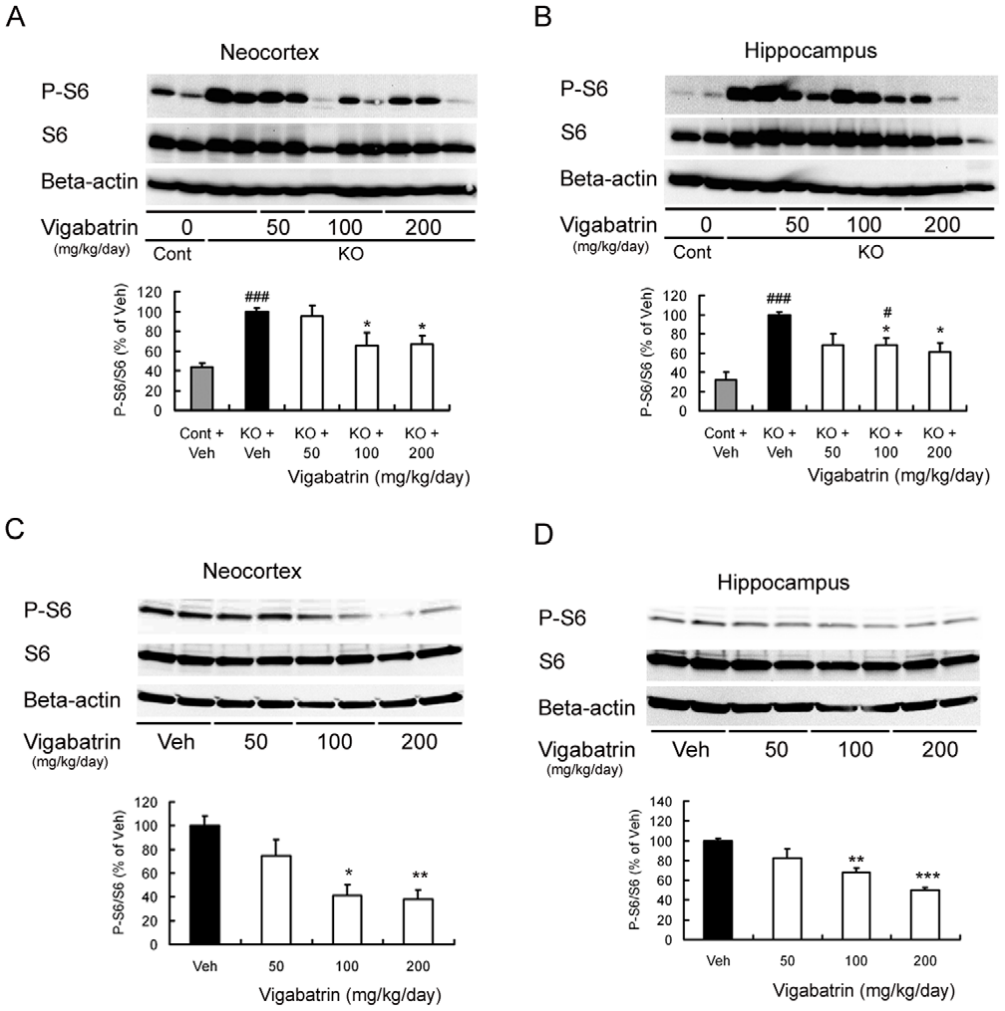
VGB decreased activation of the mTOR pathway in vivo. (A, B) Western blotting shows P-S6 (Ser240/244), total S6, and beta-actin expression in neocortex (A) and hippocampus (B) of control mice and *Tsc1* ^GFAP^CKO mice treated with vehicle or VGB at daily doses of 50, 100 and 200 mg/kg for 1 week. Quantitative summary demonstrates that vehicle-treated *Tsc1* ^GFAP^CKO mice have significantly increased P-S6 levels compared with control mice (# p<0.05, ### p<0.001 versus control mice by two-way ANOVA, n = 5–9 mice/group), and VGB inhibited the activation of S6 in *Tsc1* ^GFAP^CKO mice in a dose-dependent fashion (* p<0.05 versus vehicle-treated *Tsc1* ^GFAP^CKO mice by two-way ANOVA, n = 5–9/group). The ratio of P-S6/total S6 was normalized to the vehicle-treated *Tsc1* ^GFAP^CKO group. (C, D) Western blotting shows P-S6 (Ser240/244), total S6 and beta-actin expression in non-KO control mice administered vehicle or VGB at 50, 100 and 200 mg/kg/day for 1 week starting at age of three weeks. Quantitative summary demonstrates that VGB inhibited the activation of S6 in control mice in a dose-dependent fashion. The ratio of P-S6/total S6 was normalized to the vehicle-treated control group. *p<0.05, **p<0.01, *** p<0.001 versus vehicle-treated non-KO control mice by one-way ANOVA (n = 5–9 mice/group). Cont  =  control mice, KO  =  *Tsc1* ^GFAP^CKO mice, Veh  =  vehicle**.**

Consistent with previous studies [21], western blot analysis showed that the brains of vehicle-treated *Tsc1* ^GFAP^CKO mice have increased mTOR pathway activation compared to control mice, as reflected by an increase in the P-S6 (Ser240/244) to S6 ratio. VGB treatment at doses of 100 and 200 mg/kg/day decreased activation of the mTOR pathway in both neocortex (Fig. 5A) and hippocampus (Fig. 5B) by a maximum of about 40% in *Tsc1* ^GFAP^CKO mice. Similar effects of VGB were observed in non-KO control mice. VGB treatment at doses of 100 and 200 mg/kg/day decreased the activation of the mTOR pathway in both neocortex (Fig. 5C) and hippocampus (Fig. 5D) in control mice.

### VGB treatment decreased activation of the mTOR pathway in cultured astrocytes

**Figure 6:**
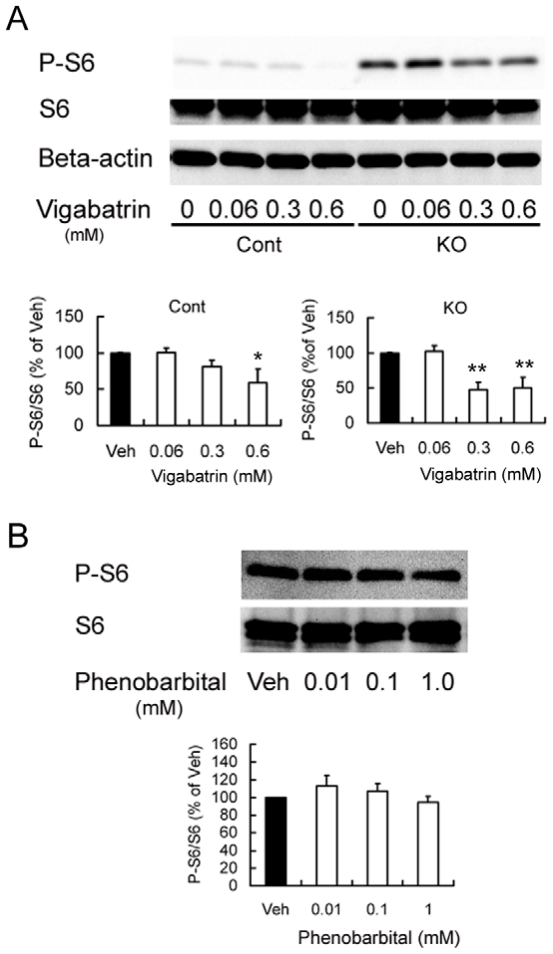
VGB decreased activation of the mTOR pathway in vitro. A) Western blotting shows P-S6 (Ser 240/244), total S6, and beta-actin expression in primary cultured astrocytes derived from *Tsc1* ^GFAP^CKO and non-KO control mice. Vehicle or VGB at a dose of 0.06, 0.3 and 0.6 mM was added to the culture medium for 16 hours. Overall, *Tsc1* ^GFAP^CKO astrocytes showed increased P-S6 expression compared with astrocytes from control mice. VGB blocked the activation of P-S6 in a dose-dependent fashion in both control and KO astrocytes. The ratio of P-S6/total S6 was normalized to the vehicle-treated control group (Cont) or vehicle-treated *Tsc1* ^GFAP^CKO group (KO). Quantitative summary demonstrates that VGB treatment at doses of 0.3 and 0.6 mM significantly inhibits the activation of P-S6 in astrocytes of both *Tsc1* ^GFAP^CKO and control mice. B) In contrast to VGB, phenobarbital had no effect on P-S6 expression in control astrocytes at doses that are effective in potentiating GABA-mediated inhibition [25]. *p<0.05, **p<0.01 versus Veh by one-way ANOVA (n = 8 mice/group). Cont  =  control mice, KO  =  *Tsc1* ^GFAP^CKO mice, Veh  =  vehicle.

Seizures themselves may directly cause acute activation of the mTOR pathway [28], [29]. In order to eliminate the possibility that VGB secondarily inhibited mTOR activity in vivo via suppression of seizure activity, the effect of VGB on mTOR activation was also tested in additional experiments in primary cultured astrocytes. VGB treatment decreased the activation of mTOR pathway in both *Tsc1* ^GFAP^CKO and control astrocytes, as reflected by the P-S6 (Ser 240/244) to S6 ratio (Fig. 6A). By comparison, the GABAergic modulator, phenobarbital had no effect on P-S6 expression (Fig. 6B).

## Discussion

VGB is particularly effective for seizures in TSC patients, but the mechanism of this unique relationship between VGB and TSC is poorly understood. In this study, we show that VGB strongly inhibits seizures in a mouse model of TSC. Consistent with its known mechanisms of action, VGB causes the expected increase in brain GABA levels in the KO mice. Furthermore, as a novel and unexpected finding, VGB inhibits mTOR pathway activity, which could represent an additional mechanism of action that may contribute to the distinctive efficacy of VGB in TSC.

VGB appears to have unique therapeutic properties in TSC for a couple of reasons. First, medical intractability is especially common in epilepsy in TSC, occurring at a much higher rate (∼65%) in epilepsy in TSC than in epilepsy overall (∼30%) [2], [7]. However, in contrast to most other antiseizure medications, VGB is particularly effective for seizures in TSC, especially infantile spasms, a typically devastating type of childhood seizure. VGB has been reported to eliminate spasms in about 95% of cases, which is much higher than all other treatments for spasms due to TSC or any other cause [8], [9]. In fact, this special relationship between VGB and TSC is one of the few documented examples of a medication having individualized efficacy for a specific epilepsy syndrome or cause of epilepsy. Furthermore, early treatment with VGB has been reported to improve the long-term outcome of neurological development and epilepsy in TSC patients [10], [11], although additional controlled clinical trials are needed to confirm these findings. In the present study in a mouse model of TSC, VGB was very effective in inhibiting epilepsy, causing almost complete suppression of seizures. The efficacy of VGB was comparatively better than other standard antiseizure medications in *Tsc1* ^GFAP^CKO mice, such as phenytoin and phenobarbital, which reduced seizure frequency by 55–68% [20]. Thus, this mouse model represents an appropriate system to investigate the mechanisms of action of VGB in TSC.

The standard, accepted mechanism of action of VGB is inhibition of the catabolism of GABA, the major inhibitory neurotransmitter in the brain [12], [13]. Irreversible inhibition of GABA transaminase by VGB leads to elevated brain GABA levels [14]. The resulting potentiation of inhibitory GABA systems in the brain is a rational explanation for the antiseizure effects of VGB. However, the effects of VGB on GABA physiology is not straightforward, as VGB causes a paradoxical reduction in evoked fast inhibitory postsynaptic potentials of neurons but instead potentiates tonic GABAergic inhibition [23]. Furthermore, enhancement of GABA signaling by increased availability of GABA may not explain the unique effectiveness of VGB for epilepsy in TSC, as other GABA-potentiating drugs, such as barbiturates and benzodiazepines, do not show comparable efficacy in TSC. In *Tsc1* ^GFAP^CKO mice, a previous study found no abnormalities in GABAergic synaptic transmission [30] and the present study demonstrates that baseline GABA levels in neocortex and hippocampus of the KO mice are similar to control mice. While *Tsc1* ^GFAP^CKO mice did show a better response to VGB in GABA levels in neocortex than control mice, this was a modest difference, and the effects of VGB on GABA levels in hippocampus were similar in control and in *Tsc1* ^GFAP^CKO mice. Thus, other mechanisms of action independent of GABA may need to be considered in order to account for the unique efficacy of VGB for seizures in TSC.

Recent evidence suggests that abnormal mTOR pathway activity is critical for epileptogenesis in TSC and that mTORC1 inhibitors, such as rapamycin, may be particularly effective therapies for epilepsy, not just as an anticonvulsant to treat seizures, but also as antiepileptogenic therapy to prevent epilepsy in TSC [16], [17]. mTOR is a protein kinase, which normally regulates a number of important functions, such as cell growth, proliferation, metabolism, and protein synthesis, many of which could affect epileptogenesis under pathological conditions. Thus, the possibility that VGB could exert at least some of its effects on epilepsy in TSC via interaction with the mTOR pathway is an intriguing, but previously untested, hypothesis. In the present study, we have provided evidence that VGB can inhibit the mTOR pathway. First, mTORC1 pathway activity, as reflected by downstream S6 phosphorylation, was decreased by VGB administered to *Tsc1* ^GFAP^CKO mice in vivo. It is important to note that other signaling pathways, such as ribosomal s6 kinases (RSK) and mitogen-activated protein (MAP)/extracelluar-signal-regulated (ERK) kinases, may also modulate S6 phosphorylation independent of mTOR. However, this mTORC1-independent phosphorylation appears to only involve the Ser235/236 phosphorylation site of S6, not the Ser240/244 site [31]. Thus, our finding that VGB decreases Ser240/244 phosphorylation supports the involvement of the mTORC1 pathway. As seizures themselves can cause mTOR activation [28], [29], one simplistic explanation for the apparent mTOR pathway inhibition by VGB in the TSC mouse model is that VGB's inhibition of seizures secondarily decreased mTOR activity. However, this possibility is basically excluded by the similar findings of mTOR pathway inhibition by VGB in normal control mice *in vivo*, as well as in cultured astrocytes from both control and *Tsc1* ^GFAP^CKO mice. By comparison, the GABA potentiating drug, phenobarbital, inhibits seizures, but has no effect on mTOR activity [29]. Furthermore, the inhibition of mTOR pathway activity by VGB in cultures *in vitro* also eliminates other confounding factors in the brain *in vivo*, suggesting that VGB directly inhibits the mTOR pathway.

The prototypic mTORC1 inhibitor, rapamycin, has potent effects in preventing epilepsy, prolonging survival, and blocking associated pathological and molecular changes that promote epileptogenesis in *Tsc1* ^GFAP^CKO mice, as well as in other mouse models of TSC [21], [32]–[34]. In the present study, in addition to seizures, VGB also had some inhibitory effects on glial proliferation, at least in hippocampus. As VGB inhibited P-S6 in both hippocampus and cortex, a similar effect of VGB on astrocyte number likely also occurred in neocortex, but the sample size may have been underpowered to reach statistical significance. Such effects of VGB on glial proliferation can likely be attributed to mTOR pathway inhibition. However, the effects of VGB were not as strong or extensive as rapamycin, as VGB only had modest effects on survival and glial proliferation and no effect on neuronal organization. Since VGB was effective in inhibiting seizures, this suggests that the pathological abnormalities in these mice are not secondary to ongoing seizures. As rapamycin is a very potent mTORC1 inhibitor [21], the difference in the effectiveness of rapamycin and VGB may be due to the milder suppression of mTOR activity by VGB in vivo, which maximized at approximately 60% of the levels of vehicle-treated *Tsc1* ^GFAP^CKO mice (Fig. 5A, B). Given these differences between VGB and rapamycin, it is possible that VGB does not directly inhibit the mTOR kinase itself like rapamycin, but may also involve other components of the mTOR pathway. Future studies are needed to determine the specific molecular mechanisms by which VGB regulates the mTOR pathway.

Other limitations of this study include issues intrinsic to the mouse model. While there are now a variety of animal models of TSC, involving inactivation of *Tsc1* or *Tsc2* at different developmental time points and in different subsets of brain cells, there is no perfect model that recapitulates all neurodevelopmental features of TSC. *Tsc1* ^GFAP^CKO mice involve primarily inactivation of *Tsc1* in glial cells, although a subset of neurons is also affected. The mechanism of action of VGB in TSC may depend on the cell type(s) affected, but this issue is not addressed with this one model of TSC. In addition, in patients with TSC, VGB is most effective against infantile spasms. Neither *Tsc1* ^GFAP^CKO mice nor any other animal model of TSC have been documented to have spasm-like seizures. Interestingly, however, rapamycin has been shown to selectively suppress spasms in a non-TSC rat model of infantile spasms [35]. Finally, the present study has not determined the relative contribution of GABA potentiation and mTOR pathway inhibition in decreasing seizures. Future studies need to define in more detail the specific cell types, seizure types, and specific mechanisms involved in VGB's effect in TSC. Despite these current limitations, the present study is significant in identifying a potential novel mechanism of action of an antiseizure medication involving the mTOR pathway. This interaction of VGB with the mTOR pathway may account for the unique efficacy of this drug for a common genetic epilepsy.
